# Late-Onset Isolated Drain Site Metastasis in Colorectal Cancer Managed by a Multidisciplinary Approach

**DOI:** 10.7759/cureus.92485

**Published:** 2025-09-16

**Authors:** David Chapman, Manaswini Krishnakumar, Adeel Shamim, Aswanth Reddy

**Affiliations:** 1 Family Medicine, Mercy Hospital, Fort Smith, USA; 2 Internal Medicine, Saint Vincent Hospital, Worcester, USA; 3 Surgery, Mercy Hospital, Fort Smith, USA; 4 Hematology and Oncology, Mercy Clinic, Fort Smith, USA

**Keywords:** colorectal cancer, delayed metastasis, genomic class switch, subcutaneous metastasis, tumor seeding

## Abstract

Colorectal cancer (CRC) is one of the most common cancers globally and a leading cause of cancer-related deaths. Over the past decade, our understanding of CRC has significantly evolved, particularly in the areas of genomic prognostication and targeted therapies. However, surgical management beyond the initial resection of the primary tumor remains an area of ongoing discussion and research.

We report a case of a 51-year-old man with metastatic CRC and liver metastases, treated with right hemicolectomy and chemotherapy, achieving complete remission. Two years later, he developed an isolated subcutaneous recurrence at the prior surgical drain site. Microsatellite instability status changed from primary to recurrent lesions. Surgical resection achieved negative margins, and the patient has since remained disease-free without additional therapy.

This rare presentation challenges traditional models of CRC metastasis and highlights the potential role of lymphatic dissemination, tumor dormancy, or surgical seeding.

## Introduction

Colorectal cancer (CRC) is widely recognized as a disease that predominantly affects older individuals, often originating as a polyp and influenced by several well-established risk factors. Over the past decade, our understanding of CRC has significantly evolved, particularly in the areas of genomic prognostication and targeted therapies [1]. However, surgical management beyond the initial resection of the primary tumor remains an area of ongoing discussion and research. In this case report, we examine the clinical course of a patient who defied conventional metastatic understanding and prognostic outcomes. By integrating current research with our patient’s unique trajectory, we aim to contribute to the ongoing scientific discourse on CRC, particularly in the context of surgery for isolated metastatic disease.

## Case presentation

**Figure 1:**
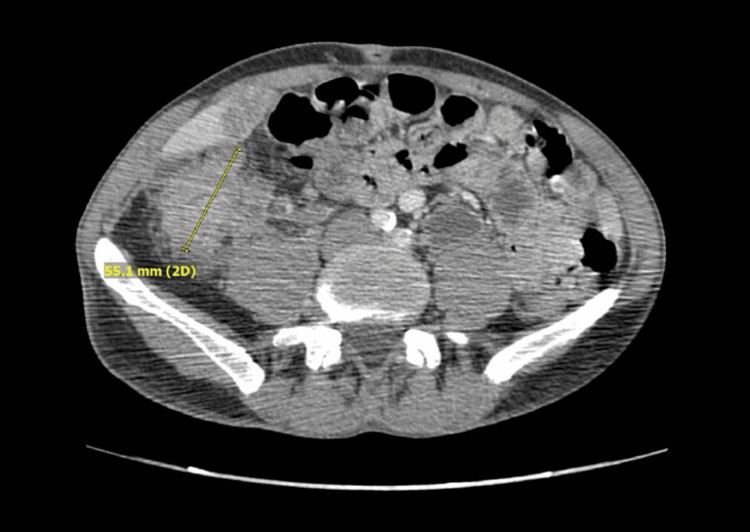
Initial CT abdomen showing a large right-sided colon mass, 06/4/6021

**Figure 2:**
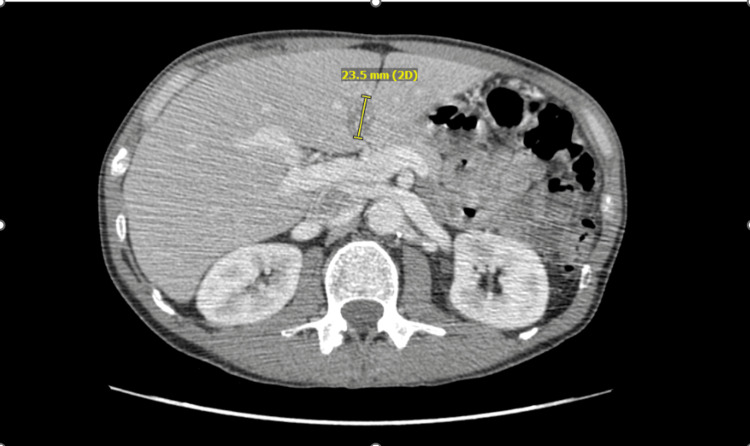
Initial CT abdomen with liver metastasis seen, 06/4/6021

Our patient, a 51-year-old man with a significant smoking history, was admitted in June 2021 with symptoms of a small bowel obstruction. A computerized tomography (CT) scan of the abdomen and pelvis revealed a mass in the cecum causing a high-grade obstruction (Figure 1), along with multiple liver lesions (Figure 2) and enlarged pericecal lymph nodes. A subsequent urgent colonoscopy confirmed the presence of an ascending colon mass, which was biopsied and identified as moderately differentiated adenocarcinoma.

**Figure 3:**
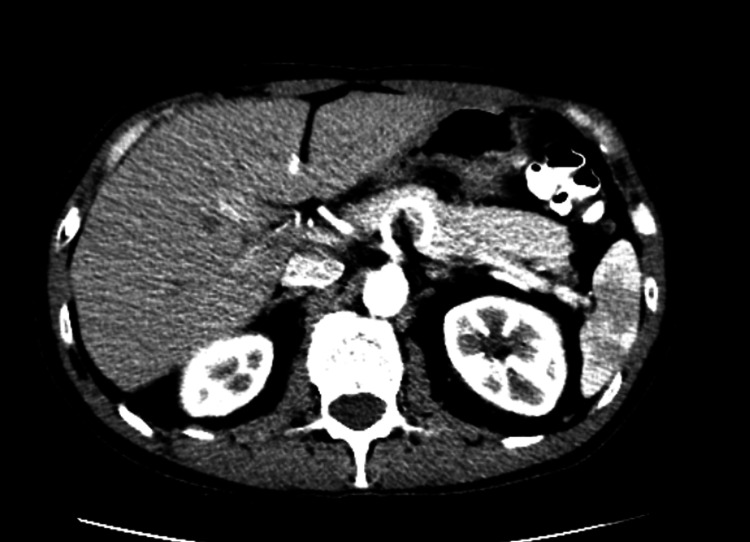
Surveillance CT abdomen pelvis showing resolution of liver metastasis, 07/12/2022

The patient immediately underwent a right extended cecal colectomy and biopsy of liver metastatic lesions. Pathological evaluation identified a 5.5 cm tumor in the cecum and proximal right colon with full-thickness extension into the pericolonic fat but negative surgical margins and no evidence of ulceration or perforation. The liver biopsy and two lymph nodes confirmed metastatic adenocarcinoma consistent with a colorectal primary, resulting in a final staging of T3 N1 M1. Next-generation sequencing revealed microsatellite stability (MSS), no RAS mutations, and equivocal HER2 expression by immunohistochemistry. Further staging evaluation showed no evidence of metastatic disease in the chest. Postoperatively, the patient completed 12 cycles of modified FOLFOX (5-fluorouracil, leucovorin, and oxaliplatin) chemotherapy with bevacizumab. He tolerated treatment well and a follow-up positron emission tomography (PET) scan post-chemotherapy showed no evidence of residual disease. Serial CT scans over the subsequent 18 months demonstrated no signs of metastatic recurrence with resolution of liver metastasis (Figure 3).

**Figure 4:**
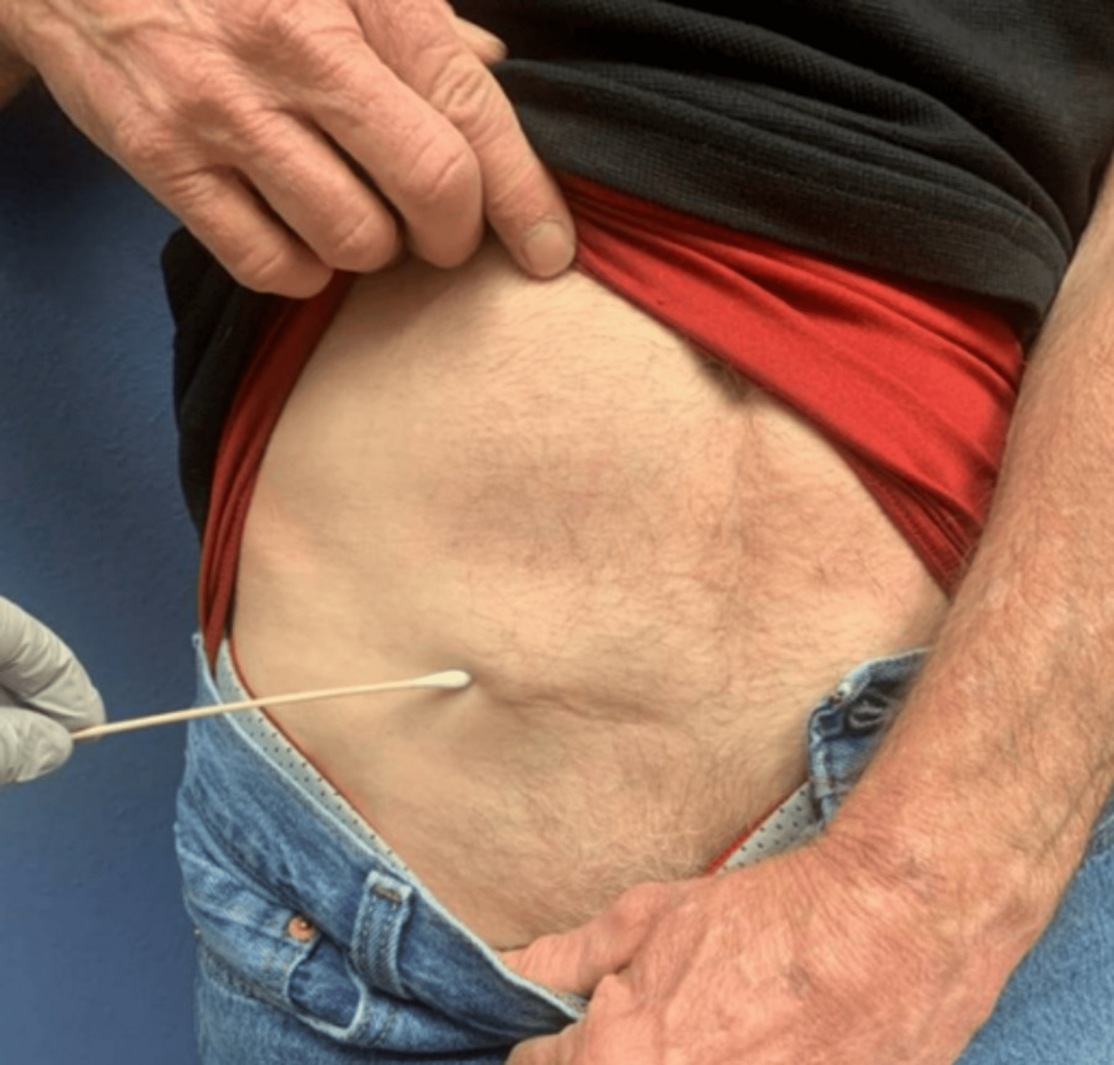
Subcutaneous mass at the previous drain site

**Figure 5:**
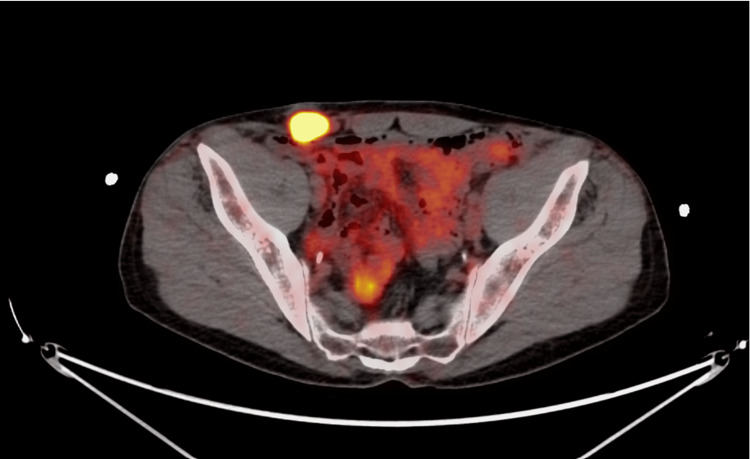
PET-CT confirming increased FDG activity at the subcutaneous mass FDG: Fluorodeoxyglucose

Two years post-treatment, a rising carcinoembryonic antigen (CEA) level was noted. On physical exam, a small subcutaneous nodule was noted in the right lower quadrant adjacent to the surgical scar from the previous drain site (Figure 4). A subsequent CT abdomen and pelvis and PET revealed a 2.4 cm subcutaneous mass in the right lower quadrant (Figure 5). A biopsy confirmed poorly differentiated adenocarcinoma, with negative CK7 and positive CDX2 and partial CK20 expression, most consistent with colorectal origin. The tumor was surgically resected with negative margins. Notably, the tumor involved the right spermatic cord, necessitating an orchiectomy. This metastatic lesion was confirmed to be located at the site of the prior abdominal drain placement two years ago. Next-generation sequencing on the drain site specimen showed negative RAS, and BRAF mutations but IHC confirmed loss of MLH1 and MSH2 proteins, confirming microsatellite instability (MSI). Post-surgery PET imaging showed no further metastatic disease, and the CEA level normalized. The patient did not receive adjuvant therapy following the second surgery and has since remained under oncologic surveillance. A PET scan two years confirmed showed no evidence of disease.

## Discussion

CRC has several well-established risk factors, including age >50, low-fiber and red meat-heavy diets, type 2 diabetes, smoking, alcohol use, ethnicity (American Indian, Alaska Native, and Eastern European Jewish descent), inflammatory bowel disease, prior radiation exposure, and hereditary syndromes such as Lynch syndrome and familial adenomatous polyposis [1]. Globally, CRC is the third most common cancer and the second leading cause of cancer-related deaths, with most fatalities resulting from metastatic disease [2].

Common metastatic sites for CRC include the liver (most frequently), lungs, and peritoneum [3]. Our patient had two primary risk factors at presentation: age and a significant smoking history. The site and nature of his metastatic recurrence raise intriguing questions about potential mechanisms of disease spread. Unlike typical peritoneal carcinomatosis, his recurrence was a solitary, well-localized mass appearing more than two years after initial treatment, situated precisely at the site of a previous abdominal drain.

Lenos et al. classify CRC into four consensus molecular subtypes (CMS): CMS1: Characterized by high immune activation, BRAF V600E mutations, and MSI tumors, more commonly found in right-sided CRCs; CMS2: Epithelial tumors with high WNT and MYC activity with high somatic copy number alterations (SCNA); CMS3: Epithelial tumors with mixed MSI status, KRAS mutations, and low SCNA; CMS4: Tumors with strong TGF-β activation, high SCNA, immune suppression, and stromal invasion, are associated with the worst survival outcomes [4].

Lenos et al. also found that CRC with peritoneal metastases (CRC-PM) were overwhelmingly classified as CMS4 (82.6%), while CRC with liver metastases (CRCLM) were more frequently CMS2 (>60%). Additionally, moesin, a key protein in peritoneal dissemination, was highly expressed in peritoneal metastases but not in liver lesions [4].

Our patient’s initial tumor characteristics suggest a CMS2 classification with MSS status and lack of RAS mutations. However, his recurrent lesion displayed features consistent with CMS1 (MSI status), raising the possibility of a class switch over time, a phenomenon documented in prior studies [5]. The delayed emergence of a solitary metastatic lesion at a prior drain site suggests either lymphatic migration of tumor cells or an exceptionally delayed surgical spillage event, both of which merit further investigation. This case emphasizes the critical importance of serial molecular profiling, as we confirmed a class switch in the metastatic tumor in our patient, and MSI status confirms a better prognosis and responses with checkpoint inhibitor therapy.

Treatment implications

Molecular profiling plays a crucial role in CRC management, guiding targeted therapy selection. While our patient lacked mutations that would warrant anti-EGFR (cetuximab, panitumumab) or immune checkpoint inhibitors (pembrolizumab) at the initial presentation, he responded remarkably well to chemotherapy (mFOLFOX) and surgical resection for isolated recurrent metastatic disease. The BECOME trial demonstrated resectability, improved progression-free survival (9.5 vs. 5.6 months), and median overall survival (25.7 vs. 20.5 months) with the addition of bevacizumab to mFOLFOX in RAS-mutant CRC-LM [6]. The CRYSTAL trial similarly showed survival benefits with cetuximab plus FOLFIRI in KRAS wild-type CRC with metastasis [7]. However, our patient’s molecular profile did not support these targeted therapies, reinforcing the importance of individualized treatment strategies.

Surgery for isolated metastasis

Metastases are an important consideration that impacts the prognosis of CRC. Death from CRC frequently results from manifestations of recurrent local or metastatic disease following initial curative therapy [8]. Because of its anatomical proximity to the portal circulation, the liver is the most frequent site of metastatic spread, followed by the peritoneum and lungs. Liver metastasis accounts for approximately 15-25% of CRC metastases at the time of primary diagnosis [9]. Unusual sites of recurrence question the conventional hypothesis and require a thorough investigation of alternative dissemination mechanisms. More than two years after the initial tumor was removed, our case offers an interesting example of a recurrence which was a solitary, well-localized mass, situated peculiarly at the site of a previous abdominal drain. Post resection, peritoneal carcinomatosis spread is either by direct invasion, peritoneal seeding, or hematogenous [10]. In this case, the site of recurrence challenges the conventional understanding of surgical spillage and suggests alternative mechanisms of tumor cell dissemination.

Tumor recurrence at a surgical drain site long after the initial treatment is rare and uncommon, with every report in the literature reviewed being in patients without metastatic disease and with recurrences much earlier [11]. Unless there was a period of dormancy before reactivation, the likelihood of direct intraoperative seeding is reduced, given our patient's prolonged period of being disease-free. Since lymphatic spread in CRC is frequently unpredictable, an isolated recurrence at an unrelated site could indicate an alternate, unfamiliar tumor cell migration pathway. This presents important questions regarding how recurrence patterns are shaped by lymphatic remodeling, tumor dormancy, and the postoperative microenvironment.

When CRC metastasizes to distant organs, usually multiple sites are involved, and treatment consists primarily of systemic chemotherapy and supportive care [12]. However, evidence indicates that certain patients with isolated metastatic lesions could benefit from more aggressive surgical treatment. In patients with isolated oligometastatic liver metastasis, surgical resection can provide a five-year survival rate of 58% in selected cases [13]. Pulmonary metastasectomy in patients with oligometastatic disease has been shown to improve survival rates by approximately 40% [14]. Patients reported in the literature who had drain site metastasis were all non-metastatic diseases at presentation, and they all had perforation contrary to our patient’s presentation [11]. Our patient had metastatic disease at presentation without perforation, had complete remission with chemotherapy, and presented with isolated drain site metastasis after two years of disease-free survival. This case supports the rationale for a personalized treatment approach, suggesting that surgical resection can provide significant disease control for select patients when feasible. It also highlights the necessity of ongoing oncological monitoring, as early detection of isolated recurrences allows for curative interventions, notably in highly aggressive unconventional recurrence cases such as our patient.

This case not only enhances our understanding of unusual recurrence patterns in CRC but also emphasizes the importance of a multimodal strategy of treatment. The late appearance of an isolated metastatic lesion in an atypical location indicates that traditional models of CRC spread may not fully explain the complexities of tumor dissemination. Additionally, the noted molecular changes in the primary vs the recurrence tumor point to the need for further research into the molecular evolution of metastatic lesions.

## Conclusions

Our patient, diagnosed with CRC with liver metastases in June 2021, underwent hemicolectomy, liver resection, and chemotherapy. He remained disease-free for over two years before developing an isolated recurrence at a prior drain site, necessitating surgical resection. Despite his advanced disease at presentation, he has significantly exceeded conventional survival expectations, emphasizing the potential impact of aggressive surgical management in select cases. This case underscores the evolving understanding of CRC metastasis, highlights the need for continued investigation into mechanisms of tumor spread and molecular class switching, and underscores the benefits of surgical interventions in long-term survival.
